# Mitochondrial DNA mutations in oxyphilic and chief cell parathyroid adenomas

**DOI:** 10.1186/1472-6823-7-8

**Published:** 2007-10-04

**Authors:** Jessica Costa-Guda, Takehiko Tokura, Sanford I Roth, Andrew Arnold

**Affiliations:** 1 Center for Molecular Medicine, University of Connecticut School of Medicine, 263 Farmington Ave, Farmington, Connecticut 06030-3101, USA; 2 Department of Pathology, Harvard Medical School and Massachusetts General Hospital, Boston, Massachusetts 02114, USA

## Abstract

**Background:**

The potential pathogenetic significance of mitochondrial DNA (mtDNA) mutations in tumorigenesis is controversial. We hypothesized that benign tumorigenesis of a slowly replicating tissue like the human parathyroid might constitute an especially fertile ground on which a selective advantage conferred by mtDNA mutation could be manifested and might contribute to the oxyphilic phenotype observed in a subset of parathyroid tumors.

**Methods:**

We sought acquired mitochondrial DNA mutations by sequencing the entire 16.6 kb mitochondrial genome of each of thirty sporadic parathyroid adenomas (18 chief cell and 12 oxyphil cell), eight independent, polyclonal, parathyroid primary chief cell hyperplasias plus corresponding normal control samples, five normal parathyroid glands, and one normal thyroid gland.

**Results:**

Twenty-seven somatic mutations were identified in 15 of 30 (9 of 12 oxyphil adenomas, 6 of 18 chief cell) parathyroid adenomas studied. No somatic mutations were observed in the hyperplastic parathyroid glands.

**Conclusion:**

Features of the somatic mutations suggest that they may confer a selective advantage and contribute to the molecular pathogenesis of parathyroid adenomas. Importantly, the statistically significant differences in mutation prevalence in oxyphil vs. chief cell adenomas also suggest that mtDNA mutations may contribute to the oxyphil phenotype.

## Background

Single parathyroid adenomas, which cause approximately 85% of the cases of primary hyperparathyroidism [1] are well-differentiated, benign, clonal tumors, which produce hypercalcemia through excessive secretion of parathyroid hormone (PTH). Activation of the *cyclin D1 *oncogene and inactivation of the *MEN1 *tumor suppressor gene are established pathogenetic contributors, but understanding of molecular pathogenesis in this disease remains incomplete. The mitochondrial genome has been identified as a possible target for somatic mutations that may promote tumorigenesis [2-10]. Mitochondrial abnormalities, including changes in structure, number, and respiratory enzyme components and transport systems, have been observed in many cancers. Homoplasmic somatic mutations of the mitochondrial genome have also been identified in many tumor types [2-10]. However, the pathogenetic significance of the mutations detected in those tissues is unclear.

The human parathyroid is an intrinsically low-turnover tissue [11], as such, benign tumors arising from this tissue have relatively low proliferative rates. The mitochondrial genome is an especially attractive potential target for mutations that could drive tumorigenesis in such a tissue. Mitochondrial DNA replicates frequently and independently of the nuclear genome [12], and over many years the potential for enhanced DNA damage from locally generated reactive oxygen species (ROS) may further enhance the accumulation of somatic mutations in the mitochondrial genome.

**Figure 1:**
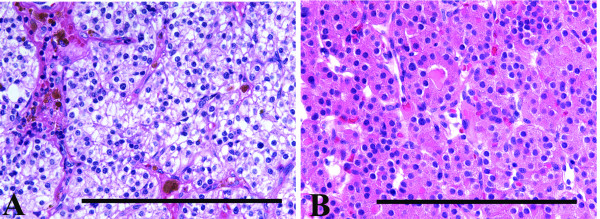
The chief cells of an adenoma (A) have an amphophilic, vacuolated cytoplasm, which on electron microscopic examination is composed of sparse mitochondria and the usual organelles associated with protein synthesis and secretion, glycogen and lipid [30]. Typical oxyphil cell adenoma (B) composed of large cells with a brightly eosinophilic granular cytoplasm, which on electron microscopic examination consists of densely packed mitochondria. The bar represents 1 mm.

In many endocrine organs, especially the parathyroid glands, with age, increasing numbers of oxyphil cells appear [13,14]. The oxyphil cells of normal parathyroid glands have a characteristic eosinophilic granular cytoplasm (Figure 1) which on electron microscopic examination is filled with abnormally shaped mitochondria [15,16]. In contrast to the chief cells, the oxyphil cells in normal glands lack the organelles associated with protein synthesis and secretion, and lack immunohistochemically identifiable chromogranin and PTH [16]. The majority of parathyroid adenomas are composed predominately of chief cells (Figure 1), however there is a subset of hyperfunctioning parathyroid adenomas that contain greater than 90% oxyphil cells and are classified as oxyphil adenomas These tumors, in addition to having a cytoplasm filled with abnormal mitochondria, contain the organelles of protein synthesis and secretion and contain histochemically identifiable PTH. While the molecular basis for this phenotypic change is unknown, it has been hypothesized that mitochondria may accumulate in oxyphil cells due to abnormalities of the mitochondrial genome[17], supported by the finding of increased numbers of oxyphil cells in normal glands with increasing age, analogous to increased mtDNA damage that occurs with aging [16].

We therefore sought acquired somatic mutations of the mitochondrial genome in both typical chief cell parathyroid adenomas and oxyphil cell parathyroid adenomas using an especially rigorous approach, and compared these findings with the mitochondrial genomes of normal and hyperplastic parathyroid glands.

## Methods

### Patient and tumor samples

Tissue samples were obtained, with Human Studies Institutional Review Board approval, from thirty-four patients who had undergone parathyroidectomy for management of primary hyperparathyroidism. Thirty samples were surgically and pathologically proven to be single parathyroid adenomas with no malignant features; of these, eighteen were determined to be typical chief cell adenomas and twelve were determined to be oxyphil adenomas, defined as containing at least 90% oxyphil cells. Eight samples from four patients were consistent with primary chief cell hyperplasia, involving multiple glands. None of the patients from whom tissue was obtained had a known history of head or neck irradiation, or a history of familial hyperparathyroidism or multiple endocrine neoplasia. One patient with primary chief cell hyperplasia had a history of lithium use. After surgical removal, tissues were dissected and quickly frozen in liquid nitrogen before being stored at -80°C. Peripheral blood leukocyte samples were obtained from the same patients, to serve as non-parathyroid germline controls. Also included in this study were five normal parathyroid glands and one normal thyroid gland from five patients with primary hyperparathyroidism due to a single adenoma. One normal parathyroid gland and a normal thyroid gland were obtained on autopsy. The remaining normal parathyroid glands were obtained from patients who had the glands removed incidental to an operation for thyroid disease.

### Mitochondrial Genome Sequencing

Genomic DNA was extracted from each sample using either proteinase K digestion for surgical samples or sucrose gradient centrifugation for blood samples, followed by phenol-chloroform extraction and ethanol precipitation.

The entire 16.6 kb mitochondrial genome from each parathyroid sample was amplified and sequenced. To avoid amplification of nuclear mitochondrial pseudogenes, the mitochondrial genome was amplified as per Polyak et al [8], as 8 overlapping 1–3 kb PCR products using a step down PCR protocol or as 11 overlapping PCR products described as follows: PCR reactions were performed in 20 μl reaction volume including 25 ng DNA, 20 pmol of each primer, 200 μM each dNTP, 1.25 U of Amplitaq Gold DNA Polymerase (Applied Biosystems, Foster City, CA) and 1.5 mM magnesium chloride; thermocycling consisted of an initial denaturation step of 95°C for 10 minutes, 35 cycles of 95°C for 30 seconds, 55°C for 30 seconds, and 72°C for 3 minutes, and a final extension step at 72°C for 10 minutes. For difficult to amplify templates, additional PCR primers were designed and are available on request. PCR products were either gel purified using the Qiagen gel extraction kit (Qiagen, Valencia, CA), or 10 U Exonuclease I and 1 U Shrimp Alkaline Phosphatase (Amersham Pharmacia Biotech, Piscataway, NJ). Purified PCR products were subjected to automated sequencing using the Big Dye Terminator Cycle Sequencing Ready Reaction Kit (Applied Biosystems). We also made and utilized additional sequencing primers, sequences available on request. Sequencing products were purified through Sephadex G-50 columns (Sigma-Aldrich, St. Louis, MO), and electrophoresed on 4.75% Long Ranger gels (BioWhittaker Molecular Applications, Rockland, ME) on an ABI Prism 377 Sequencer (Applied Biosystems).

To determine if sequence changes identified in patients with hyperparathyroidism were somatic and/or clonal, the corresponding mitochondrial genomic regions from the patient's normal control peripheral blood leukocytes were also sequenced as above. Resulting sequence data files were analyzed and compared with the standard Anderson mtDNA sequence using the sequence analysis programs Sequencing Analysis and Auto Assembler (Applied Biosystems). Deviations from the standard sequence were compared with the on-line Mitomap database of previously reported mtDNA mutations and polymorphisms [18]. All apparent somatic clonal mutations were confirmed by sequencing products of independent PCR reactions, and by sequencing the opposite DNA strand.

## Results

A total of 393 homoplasmic sequence variants were identified (8–45 variants per sample) as compared to the standard Anderson mtDNA sequence [19]. Of the 393 variants, 375 were single base pair substitutions, 13 were single base pair insertions or deletions (4 deletions and 9 insertions), 2 were 2 base pair deletions, 2 were 2 base pair insertions and 1 was an 8 base pair deletion. Evaluation utilizing the mitochondrial database, Mitomap [18], indicated that of the 393 variants, 117 were in non-coding regions, 244 were in protein coding genes and 32 were predicted to affect rRNA or tRNA. Novel sequence variants are shown in Table 1.

**Table 1:** Novel germline sequence variants identified by this study

**Nucleotide position**	**Base Change**	Region Affected	Expected Protein Change
1007	g-a	12s rRNA	n/a
1339	g-c	12s rRNA	n/a
2218	c-t	16s rRNA	n/a
2639	c-t	16s rRNA	n/a
3159	ins t	16s rRNA	n/a
3198	a-c	16s rRNA	n/a
3511	a-g	ND1	69 Thr-Ala
3719	a-g	ND1	138 Gln-Arg
3826	t-c	ND1	no change
3867	c-t	ND1	no change
4296	t-c	Ile tRNA	n/a
4674	a-g	ND2	69 Ile-Val
4718	a-g	ND2	no change
4735	c-a	ND2	89 Thr-Arg
5238	c-t	ND2	no change
5250	t-c	ND2	no change
5318	c-t	ND2	no change
5463	c-t	ND2	332 Leu-Phe
6026	g-a	COI	42 Gly-Asp
6339	a-g	COI	146 Thr-Ala
6425	t-c	COI	no change
6570	g-t	COI	223 Ala-Ser
6656	c-t	COI	no change
6775	a-c	COI	291 His-Pro
7295	a-g	COI	no change
7744	t-c	COII	no change
7963	a-g	COII	no change
7966	c-t	COII	no change
8395	c-t	ATPase8	no change
8515	c-t	ATPase8	no change
8602	t-c	ATPase6	26 Phe-Leu
8953	a-g	ATPase6	143 Ile-Val
8987	t-g	ATPase6	154 Met-STOP
9088	t-c	ATPase6	188 Ser-Pro
9210	a-g	COIII	2 Thr-Ala
9233	t-c	COIII	no change
9426	c-t	COIII	74 Pro-Ser
9555	c-a	COIII	117 Pro-Thr
9614	a-t	COIII	no change
9656	t-c	COIII	no change
10152	g-c	ND3	32 Glu-Gln
10325	g-a	ND3	no change
10754	a-c	ND4L	no change
10775	g-a	ND4	6 Val-Ile
10973	c-a	ND4	72 Leu-Ile
11151	c-t	ND4	131 Ala-Val
11354	t-c	ND4	199 Tyr-His
11566	a-g	ND4	no change
11617	t-c	ND4	no change
11911	c-a	ND4	no change
11928	a-g	ND4	390 Asn-Ser
12879	t-c	ND5	no change
12953	c-t	ND5	206 Ala-Val
12954	t-c	ND5	no change
12976	c-g	ND5	214 Leu-Val
13129	c-t	ND5	265 Pro-Ser
13132	c-t	ND5	no change
13260	t-c	ND5	no change
13264	c-t	ND5	no change
13392	t-c	ND5	no change
13422	a-g	ND5	no change
13708	g-a	ND5	458 Ala-Thr
13743	t-c	ND5	no change
14364	g-a	ND6	no change
14581	t-c	ND6	no change
14694	c-g	Gln tRNA	n/a
14814	c-g	CYTB	23 Thr-Ser
14869	g-c	CYTB	no change
15148	g-a	CYTB	no change
15172	g-a	CYTB	no change
15391	c-t	CYTB	no change
15544	c-a	CYTB	no change
15629	t-c	CYTB	no change
15661	c-t	CYTB	no change
15697	t-g	CYTB	317 Phe-Leu
15709	c-g	CYTB	321 Ser-STOP
15783	c-t	CYTB	346 Pro-Leu
16478	c-t	non-coding	n/a
16521	a-c	non-coding	n/a

**Figure 2:**
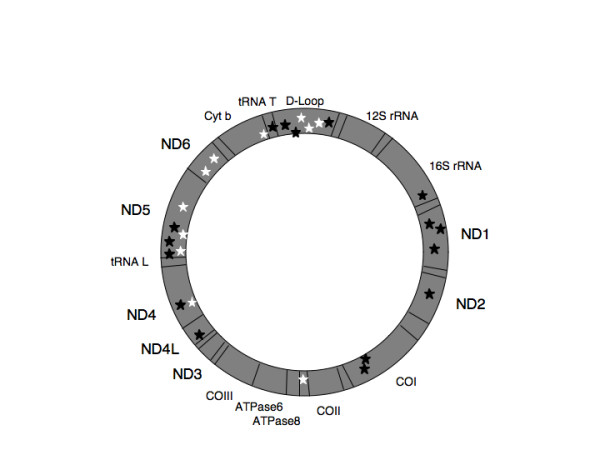
Schematic diagram of the mitochondrial genome including locations of the somatic mutations identified in this study. Locations of somatic mutations in parathyroid chief cell and oxyphil cell adenomas are indicated by white and black stars, respectively.

Eleven somatic mutations were identified in 6 of 18 chief cell adenomas (Table 2 and Figure 2). Two tumors contained mutations that were predicted to result in early stop codons in either the *ND4 *or *ND6 *genes, both subunits of mitochondrial Complex I. Three tumors contained mutations that were predicted to result in non-synonymous changes in subunits of either Complex I or Complex III. Two tumors contained mutations only in the highly polymorphic, but non-coding D-loop region.

**Table 2:** Summary of somatic mutations identified in parathyroid adenomas

Tumor ID	Tumor Type	Mutation	Gene affected	Expected Protein Change	Previously seen?
1	Oxyphil cell	12631T>C	ND5	99Ser>Pro	normal variant
2	Oxyphil cell	3173G>A	16S rRNA		novel
		6869C>T	CO1	no change	somatic mutation-cancer cell line
		12425delA	ND5	30Asn>Frameshift	novel
3	Oxyphil cell	559G>A*	D-loop		normal variant
		7028T	CO1	no change	normal variant
4	Oxyphil cell	3572insC	ND1	89Leu>Frameshift	novel
5	Oxyphil cell	15924A>G	Thr tRNA		LIMM
6	Oxyphil cell	304C>T	D-loop		normal variant
		11038delA	ND4	93Lys>Frameshift	novel
7	Oxyphil cell	310insC	D-loop		normal variant
8	Oxyphil cell	3566insC	ND1	87Thr>Frameshift	novel
9	Oxyphil Cell	4172T>C	ND1	289Leu>Pro	novel
		5026A>G	ND2	186His>Arg	somatic mutation-oral cancer
		10522G>A	ND4L	18Gly>Glu	novel
		12372G>A	ND5	no change	normal variant
10	Chief Cell	11038delA	ND4	93K>Frameshift	novel
		13577T>C	ND5	414Ile>Thr	novel
		14386T>C	ND6	no change	normal variant
11	Chief Cell	16311T>C	D-loop		normal variant
12	Chief Cell	12382A>G	ND5	16Ile>Val	novel
13	Chief Cell	15578T>C	CYTB	278Tyr>His	novel
14	Chief Cell	8281del8	non-coding		novel
		12753A>G	ND5	no change	novel
		14488delT	ND6	62Gly>Frameshift	novel
		16519T>C	D-loop		normal variant
15	Chief Cell	253C>A	D-loop		somatic mutation

* indicates that this mutation appeared to be heteroplasmic

LIMM-Lethal Infantile Mitochondrial Myopathy

Sixteen somatic mutations were identified in 9 of 12 oxyphil adenomas. Four tumors contained mutations that were predicted to result in early stop codons in either the *ND1*, *ND4 *or *ND5 *genes, all subunits of mitochondrial Complex I. Two tumors contained non-synonymous changes in Complex I subunits, one tumor contained a mutation in *CO1 *not predicted to change the amino acid sequence and one tumor contained a mutation affecting a tRNA gene. One tumor contained a D-loop mutation only. All mutations appeared to be homoplasmic, with the exception of one, which appeared to be heteroplasmic, a G to A transition at position 559, within the D-loop region. Homoplasmy refers to the presence of a particular sequence variant or mutation in virtually every copy of the mitochondrial genome within a single tumor cell sample (and within the limits of sensitivity of the sequencing technology), while heteroplasmy refers to the presence of more than one such detectable allele [20]. Heteroplasmy within a tumor would be indistinguishable from homoplasmy in a sub-population of the tumor's cells, since each tumor's cells are being analyzed collectively rather than on an individual cell-by-cell basis.

Mutations affecting Complex I genes, which represent 38.2% of the mitochondrial genome, accounted for 55.6% of all mutations and 22.2% of mutations occurred in the highly variable D-loop region.

To determine if the observed trend towards increased numbers of likely or potentially functionally significant mtDNA mutations in oxyphil adenomas as compared to chief cell adenomas was statistically significant, Fisher's Exact test was performed yielding a significant p-value of 0.02.

No somatic mutations occurred in any of the hyperplastic parathyroid glands. The one normal parathyroid for which paired thyroid tissue was available also contained no somatic mutations. No clustering of germline sequence variants occurred in any of the samples studied.

## Discussion

Mitochondrial abnormalities have been observed in many cancers and previous studies have demonstrated altered energy metabolism in cancer cells [2-10]. Homoplasmic somatic mutations of the mitochondrial genome have been identified in various tumor types. The mitochondrial genome is an especially attractive potential target for mutations that could drive tumorigenesis in a low turnover tissue such as the human parathyroid, given the frequent replication of mitochondrial DNA, independent of the nuclear genome and the enhanced potential for mitochondrial DNA damage by ROS. One might expect an increased potential for accumulation of somatic mutations in the mitochondrial genome in such low replicative tissues as the parathyroid over many years. Our data provide initial support for these hypotheses, and suggest that other slow-growing tumors arising from low-replication tissues should be similarly analyzed.

Mitochondrial DNA mutations in parathyroid adenomas tend to affect Complex I genes more often than other tumor types and more often then expected by chance alone. Additionally, D-loop mutations appear to be underrepresented in parathyroid adenomas compared to other tumor types. In contrast to many other studies, this study rigorously examines the entire mitochondrial genome in matched tumor/normal pairs from the same individual while many analogous studies of other types of tumors focused primarily on coding regions and/or the D-loop region or used population rather than control DNA from the same patient. Due to the wide range of mutation frequencies in the literature and discrepancies in study design, statistical analyses between our data and those from other related studies cannot generally be performed. The importance of observed differences between parathyroid adenomas and other tumor types remains unclear.

Our results raise the possibility that NADH dehydrogenase subunit genes might play a key role in tumorigenesis, perhaps especially in low-replication tissues. Few studies have directly examined this issue. The contribution of specific mtDNA mutations, affecting the ATP synthase subunit 6 [21,22], to tumorigenesis has been examined, however little is known about the potential pathogenetic significance of the majority of mtDNA mutations identified to date. The contribution of specific germline sequence variants as predisposition alleles to specific tumor types has been suggested, however the majority of germline mtDNA sequence variants appear unlikely to directly contribute to tumorigenesis because of the frequency of such changes in individuals without tumors. Muller-Hocker, et al. examined respiratory chain function in normal parathyroid glands and hyperplastic parathyroids in uremic patients[23], finding defects in Complex III and IV in both normal and diseased glands. They also reported that chief cells in 20% of uremia-associated (secondary) hyperplastic parathyroid glands had defects of the respiratory chain, and that heart and muscle cells had more respiratory chain defects than parathyroid oxyphil cells. Together with reports of respiratory chain defects in normal, adenomatous, and hyperplastic parathyroid glands, the preferential clustering within NADH dehydrogenase subunit genes of the somatic mitochondrial mutations in our series of adenomas reinforces the intriguing suspicion that these mutations may be playing an integral role in parathyroid tumorigenesis. Given that mitochondria have recently been implicated in the regulation of intracellular calcium homeostasis [24,25] and apoptosis [26], and that calcium sensing and signaling are crucial to parathyroid function and regulated PTH secretion, the possible relationship between calcium homeostasis and mitochondrial complex I enzyme function deserves further scrutiny.

Our data also provide additional support for the hypothesis that mitochondria accumulate in oxyphil cells due to abnormalities of the mitochondrial genome. Previous studies of Hurthle cell [27] and other oncocytic tumors [28] have demonstrated an association between a large deletion of mtDNA (Δ4977) and oncocytic/oxyphilic tumors in comparison to non-oncocytic tumors of the same tissue type. However, given its presence in both normal and tumor cells of various tissues, the potential pathogenetic significance of such a finding remains unclear. As such, the presence or absence of this deletion was not addressed by our experimental approach and was considered beyond the scope of this manuscript. Functioning oxyphil cell parathyroid adenomas are more likely to contain somatic mtDNA mutations than chief cell adenomas and the somatic mutations in oxyphil adenomas are more likely to be functionally significant. These differences between oxyphil and chief cell adenomas are statistically significant with a p-value of 0.02 (Fisher's Exact Test). Our results show marked similarity to the recent findings in thyroid tumors by Gasparre *et al *that disruptive mutations (nonsense and frameshift mutations) in Complex I genes are associated with an oncocytic phenotype [29]. It is therefore quite plausible that these molecular genetic lesions may make key contributions to determining the oxyphilic phenotype, as mitochondria may increase their replication rate as a compensatory mechanism for altered energy metabolism due to mtDNA mutations. Further studies of normal oxyphil cells that could shed additional light on this hypothesis should be encouraged.

## Competing interests

The author(s) declare that they have no competing interests.

## Authors' contributions

JC-G participated in the design of the study, carried out molecular genetic studies and sequence analyses, and drafted the manuscript. TT participated in the design of the study and carried out molecular genetic studies and sequence analyses. SIR performed histological analyses for all tumor samples. AA participated in the design and coordination of the study, data analysis and interpretation. All authors participated in revising drafts of the manuscript, and all authors read and approved the final manuscript.

## Pre-publication history

The pre-publication history for this paper can be accessed here:


